# Up-regulated miR-133a orchestrates epithelial-mesenchymal transition of airway epithelial cells

**DOI:** 10.1038/s41598-018-33913-x

**Published:** 2018-10-19

**Authors:** Linjie Chen, Xiaobai He, Yan Xie, Yapei Huang, Dennis W. Wolff, Peter W. Abel, Yaping Tu

**Affiliations:** 1 Department of Pharmacology, Creighton University School of Medicine, Omaha, NE USA; 2 College of Biotechnology, Jiangsu University of Science and Technology, Zhenjiang, Jiangsu China; 3 Kansas City University of Medicine and Biosciences-Joplin, Joplin, MO USA

## Abstract

Dysregulation of microRNAs (miRNAs) contributes to epithelial-mesenchymal transition (EMT) of cancer, but the pathological roles of miRNAs in airway EMT of lung diseases remains largely unknown. We performed sequencing and real-time PCR analysis of the miRNA expression profile of human airway epithelial cells undergoing EMT, and revealed miR-133a to be one of the most common up-regulated miRNAs. MiR-133a was previously reported to be persistently up-regulated in airway epithelial cells of smokers. We found that mice exposed to cigarette smoke (CS) showed airway hyper-responsiveness, a typical symptom occurring in CS-related lung diseases, up-regulation of miR-133a and EMT marker protein N-cadherin in airway epithelium. Importantly, miR-133a overexpression induces airway epithelial cells to undergo spontaneous EMT via down-regulation of grainyhead-like 2 (GRHL2), an epithelial specific transcriptional factor. Loss of GRHL2 causes down-regulation of epithelial splicing regulatory protein 1 (ESRP1), a central coordinator of alternative splicing processes that are critical in the regulation of EMT. Down-regulation of ESRP1 induces isoform switching of adherens junction-associated protein p120-catenin, and leads to the loss of E-cadherin. Our study is the first to demonstrate that up-regulated miR-133a orchestrates airway EMT via alternative splicing processes, which points to novel therapeutic possibilities for the treatment of CS-related lung disease.

## Introduction

Epithelial-mesenchymal transition (EMT) is a process by which differentiated epithelial cells lose their defining characteristics and acquire mesenchymal characteristics^1^. EMT can be divided into three subtypes that are integral to development, wound healing and stem cell behavior, and contribute pathologically to fibrosis and cancer progression^1^. Reversible type I EMT occurs during embryonic development. Type II EMT happens in wound healing, and irreversibly generates fibroblasts and organ fibrosis in response to tissue injury and inflammation. Type III EMT occurs during cancer progression, including metastasis, formation of cancer stem cells or helping cancer cells escape from chemotherapy^2^. Accumulating evidence now supports the importance of Type II EMT in the pathogenesis of lung diseases such as pulmonary fibrosis, asthma and chronic obstructive pulmonary disease (COPD) during airway injury and inflammation^3–6^. Previous studies have reported that 30% of peribronchiolar fibroblast cells were derived from EMT of airway epithelial cells in pulmonary fibrosis *in vivo*^7,8^.

Many distinct molecular processes are engaged to initiate and/or complete EMT, including activation of transcription factors, changes of cell-surface proteins, reorganization and expression of cytoskeletal proteins, and production of ECM-degrading enzymes^1,6^. More recent studies have implicated microRNAs (miRNAs) in EMT progression^9–11^. MiRNAs are small single-stranded non-coding RNAs that regulate expression of 60% of human genes through base-pairing to the 3′-untranslated region (3′UTR) of target mRNAs, resulting in mRNA degradation and/or translational repression. Dysregulation of miRNAs has been found in multiple human diseases including asthma, COPD and pulmonary fibrosis^12–14^. For example, typical miR-200- and miR-205-family epithelial miRNAs are down-regulated during EMT to enhance the translation of mesenchymal transcriptional factor ZEB1 and ZEB2 mRNAs in breast cancer cells^9^. Up-regulated miR-10a during EMT would repress E-cadherin to initiate EMT in breast cancer cells^15^. However, regulation and individual pathological roles of these miRNAs in airway EMT during the progression of lung diseases remain largely unknown.

MiR-133a is a mesenchymal miRNA highly conserved in skeletal and cardiac muscle, and plays central roles in the regulation of myogenesis, muscle development and muscle remodeling. Abnormal expression of miR-133a has been observed during progression of many solid tumors^16^. For example, down-regulation of miR-133a was found in bladder and lung squamous cell carcinoma^17,18^ whereas miR-133a was selectively up-regulated in cervical cancer^19^. Up-regulation of miR-133a was also found in small airway epithelium of smokers, which persisted even after quitting smoking for 3 months^20^, but its contribution to smoking-related lung disease pathogenesis at a molecular level had not been investigated.

Herein, we report that miR-133a orchestrates EMT in airway epithelial cells. We first identified miR-133a as one of the most common up-regulated miRNAs in airway epithelial cells undergoing EMT. Change in mesenchymal protein expression levels were then evaluated in cultured cells and mice exposed to cigarette smoke (CS). Data obtained support the hypothesis that up-regulated miR-133a induces airway EMT. Further mechanistic studies revealed that miR-133a directly targets mRNA of the transcriptional factor Grainyhead-like 2 (GRHL2) to repress its protein expression. Loss of GRHL2 protein triggers isoform switching of adherens junction-associated protein p120-catenin (p120ctn) via down-regulation of epithelial splicing regulatory protein 1 (ESRP1), which in turn causes E-cadherin down-regulation, a hallmark of EMT.

## Methods

### Cells and culture

Immortalized human bronchial epithelial cell line Beas-2b (ATCC ® CRL-9609) and HEK293 (ATCC® CRL-1573) cells were from the American Type Culture Collection (Manassas, VA). Primary human bronchial epithelial cells (NHBE) were from Lonza (Walkersville, MD). Primary human lung fibroblasts were isolated and cultured from lung tissues obtained during open lung biopsy from patients at the time of death. Two primary human lung fibroblast cell lines were established by Dr. Reynold Panettieri’s laboratory (University of Pennsylvania) from two patients with brain-related disease but no history of pulmonary fibrosis^21^. The biopsies had no identifiers, and the protocols for cell isolation were approved by the university Institutional Review Boards. Mouse tracheal epithelial cells were isolated as previously described^22^. Beas-2b and NHBE cells were cultured in BEGM medium (Lonza). HEK293 and fibroblast cells were cultured in DMEM medium (Hyclone, Logan, UT) with 10% fetal bovine serum.

To enrich Beas-2b cells with spontaneous EMT, cells were grown to 50~60% confluency and then treated with 0.05% trypsin for 1 min. The detached cells were reseeded and cultured for one week. This procedure was repeated twice and the detached cells were then collected and reseeded to 12-well plates at a density of 500 cells per well. Wells of cells showing a mesenchymal phenotype were selected for further expansion and analysis. To examine TGFβ1-induced EMT, Beas-2b cells were seeded a day prior in 6 well or 12 well plates, and then stimulated at ~20% confluence without or with 5 ng/ml of recombinant human TGFβ1 (R&D Systems, Minneapolis, MN) in complete BEGM medium. Medium with or without TGFβ1 was changed every 3 days. Cells were harvested at the indicated time for RNA and protein analysis.

### Construction of plasmids

All primers are shown in Supplementary Table S1. To construct luciferase reporter plasmid containing GRHL2–3UTR-133a, the 3′UTR fragments containing miR-133a binding sites of human GRHL2 were amplified from Beas-2b cell genomic DNA with XbaI- and SacI-flanked primers and cloned into the pmirGLO reporter vector (Promega, Fitchburg, WI). To construct the GRHL2–3UTR-133a/M plasmid, miR-133a binding site mutants were generated by over-lap PCR as shown by the underlined nucleotide changes. To construct PX459-GRHL2 g1 and g2 plasmids, gDNA1 or gDNA2 oligos were inserted into PX459 plasmids (Addgene #62988) according to the published protocol^23^. Mouse ESRP1 was sub-cloned into pcDNA3.1 from the pBRIT-ESRP1 expressing vector kindly provided by Dr. Chonghui Cheng at Northwestern University)^24^. All constructs were validated by DNA sequencing.

### MicroRNA sequencing and real time PCR

Total RNA was isolated from samples with a mirVana miRNA Isolation Kit (Invitrogen). For small RNA sequencing, a Fragment Analyzer™ Automated CE System was used to check RNA quality; a NEXTflexTM Small RNA-Seq Kit v3 (Bioo Scientific #5132-05) was used with 1 µg of total RNA for the construction of small RNA sequencing libraries according to the standard Illumina protocols; Illumina Nextseq 500 was used for miRNA-sequencing at the University of Nebraska Medical Center Next Generation Sequencing Core Facility (Omaha, NE)). MiRNAs of two normal epithelial-like Beas-2b cell sub-populations and two mesenchymal-like Beas-2b/M cell sub-populations were sequenced. The sequencing data presented in this manuscript have been deposited in NCBI’s Gene Expression Omnibus and are accessible through the link (http://www.ncbi.nlm.nih.gov/geo/query/acc.cgi?acc = GSE117522). The miRNA analysis app (B&Gu @ University of Torino) in BaseSpace Sequence Hub was used for quantitative miRNA expression analysis. For real-time PCR, relative miRNA expression levels were determined by TaqMan™ MicroRNA Assays (Thermo Fisher Scientific, Waltham, MA). RUN6B and Sno202 were used as endogenous miRNA controls for human and mouse miRNA samples, respectively. The relative miR-133a expression level in a control group was adjusted to 1.

### Small RNAs and plasmids transfection

MiRNA mimics (miR mimics control: #CN-002000-01, miR-133a mimics: #C-300600-05-0002) and GRHL2 siRNA (#L-014515-02-0005) were from GE Dharmacon (Lafayette, CO). Small RNA transfection was performed with a Nucleofector® II Device (Lonza, Walkersville, MD) plus Lipofectamine® RNAiMAX Reagent (Invitrogen) according to manufacturer’s instructions for Beas-2b cells and with Lipofectamine® RNAiMAX Reagent only for NHBE cells. Plasmid transfection of Beas-2b cells was performed by Nucleofector® II Device plus Lipofectamine® 3000 Reagent (Invitrogen) according to manufacturer’s instructions.

### CRISPR/Cas9 genome editing

Beas-2b cells were mock transfected or transfected with PX459 (control) or PX459-GRHL2-g1/g2. 24 hours later, the transfected cells were selected in the full growth medium with 300 ng/ml puromycin until the mock transfected cells had no survival. At the end of selection, genomic DNA was isolated and GRHL2 gene knockout was confirmed by conventional PCR using high fidelity DNA polymerase. All primers are listed in Supplementary Table S1.

### CS exposure and airway hyper-responsiveness measurement of mice

All animal studies were carried out in accordance with recommendations in the Guide for the Care and Use of Laboratory Animals of the National Institutes of Health. The protocol was approved by the Institutional Animal Care and Use Committee at Creighton University. Mice were exposed to mainstream CS (Reference Cigarette 3R4F with a filter, University of Kentucky, Lexington, KY) using a smoking generator (Data Sciences International, St. Paul, MN). Pups on postnatal days 2–11 (PND 2–11) with their mother were exposed to 100 µg/m^3^ whole smoke (1.5 hour per exposure, 2 times per day). After recovery (PND12–59), mice were re-exposed to 150 µg/m^3^ whole smoke (3 hours exposure per day, 3 days per week) for four weeks. The control groups were exposed to air. One day after the last CS exposure, mice were anesthetized for airway hyper-responsiveness measurements as we previously reported^25^. Mice were then euthanized for immunohistochemistry analysis of lung tissues and tracheal epithelial cell isolation.

### Immunohistochemistry staining of N-cadherin on mouse lung sections

Lungs were collected and fixed in formalin. Parasagittal sections of tissue representing central and peripheral airways were embedded in paraffin, cut at 5-μm thickness, and stained for N-cadherin using the method previously described for α-smooth muscle actin immunostaining^25^.

### Western blot

Protein extracts from cultured cells were quantified using Pierce™ Coomassie (Bradford) Protein Assay Kit (Thermo Fisher Scientific, Waltham, MA) and then subjected to western blot analysis as we reported^26^. Primary antibodies against E-Cadherin (24E10 rabbit mAb #3195), N-Cadherin (D4R1H XP® rabbit mAb #13116), vimentin (D21H3 XP® rabbit mAb #5741), and Snail (C15D3 rabbit mAb #3879) were from Cell Signaling Technology (Beverly, MA). Additional sources of primary antibodies: β-actin (AC-15 mouse mAb #sc-69879) was from Santa Cruz Biotechnology (Dallas, TX); GAPDH (1E6D9 mouse mAb #60004-1-Ig) was from Proteintech Group, Inc. (Rosemont, IL); GRHL2 (mouse polyclonal, #ab88631) and ESRP1/2 (mouse mAb [23A7.C9], #ab106555) were from Abcam (Cambridge, MA); and P120ctn (mouse mAb, #610133) was from BD Transduction Laboratories™ (San Jose, CA).

### Luciferase reporter assay

HEK293 cells were transfected with control or miR-133a mimics and the pmirGLO luciferase constructs with/without insertion of the 3′UTR fragments of GRHL2 using Lipofectamine 3000 according to the manufacturer’s instructions. Twenty-four hours after transfection, cells were analyzed for luciferase activity using a Dual-Glo® Luciferase Assay kit (Promega) and Sirius luciferase assay system (Berthold, Germany). Normalized firefly luciferase activity (firefly luciferase activity/Renilla luciferase activity) for each construct was compared to that of the pmirGLO Vector no-insert control.

### Statistical analysis

Data are expressed as means ± S.E. of at least three determinations. Groups were compared using Student’s t test for unpaired observations or two-way ANOVA with the Bonferroni correction for multiple comparisons. *p < 0.05; **p < 0.01; ns, not significant.

## Results

### Identification of miR-133a as a highly up-regulated microRNA in mesenchymal-like cells derived from Beas-2b airway epithelial cells

**Figure 1:**
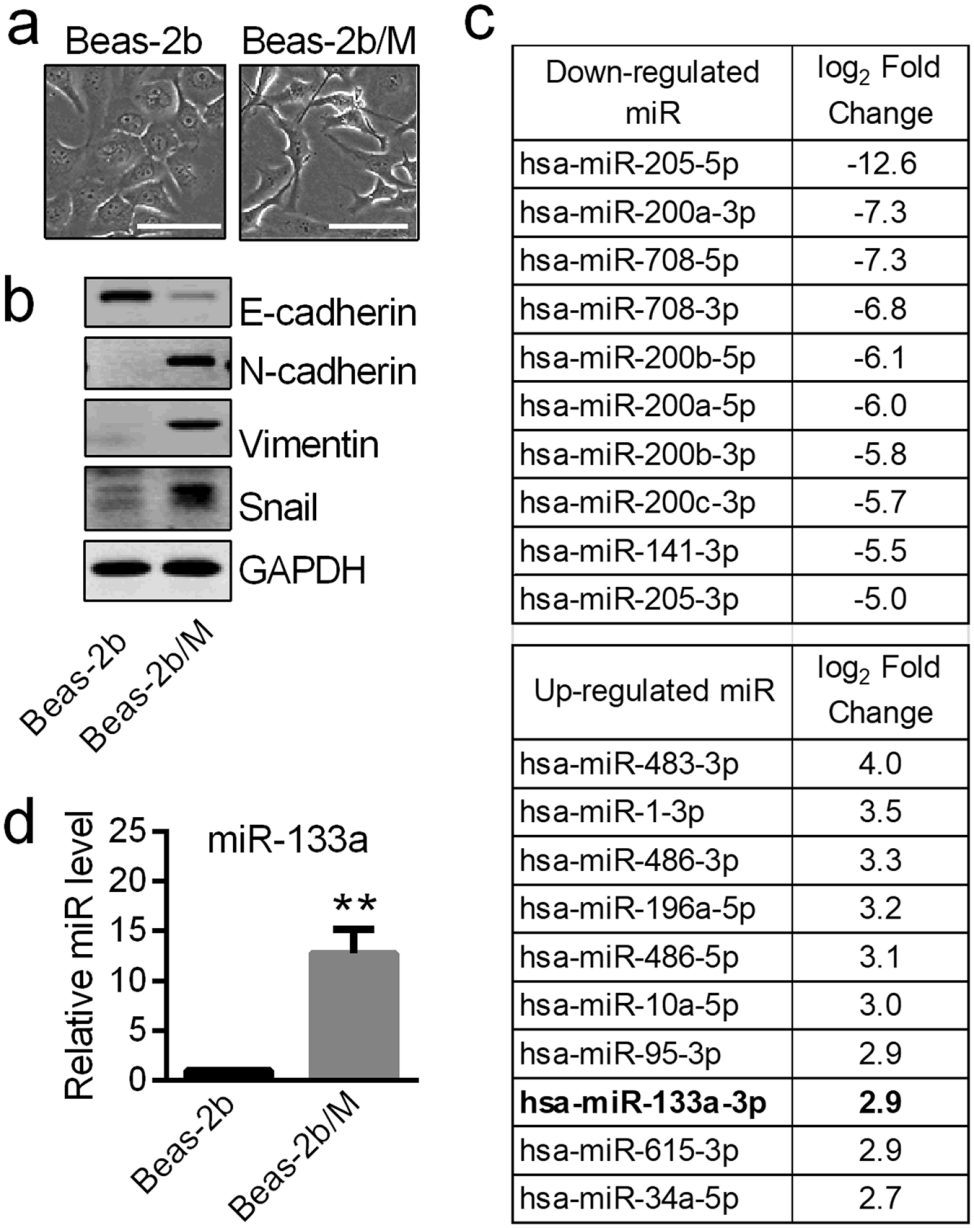
Identification of miR-133a as a candidate involved in EMT of airway epithelial cells. (**a**) Phase contrast images of Beas-2b cells with epithelial (left) or mesenchymal (right) phenotype. Control Beas-2b epithelial cells have extensive interactions with each other while Beas-2b/M cells are spindle-like and interact with each other only though focal points. Scale bars, 100 µm. (**b**) Western blot analysis of EMT associated proteins in Beas-2b and Beas-2b/M cells. Experiments were conducted at least three times, and a representative result is shown. The grouped blots were cropped from different parts of the same gel. Unprocessed original scans of the blots are shown in Supplementary Fig. S5. (**c**) MiRNA sequencing was used to profile the miRNA expression in Beas-2b and Beas-2b/M cells. The upper table lists the top ten down-regulated miRNAs and the lower table lists the top ten up-regulated miRNAs in Beas-2b/M cells compared to epithelial Beas-2b cells. Normalized log_2_ data shown are the average of duplicates. (**d**) TaqMan quantitative PCR validation of miR-133a up-regulation in Beas-2b/M cells when compared to epithelial-like Beas-2b cells. RUN6B was used as endogenous control. Data are means ± S.E. (n = 4; **P < 0.01).

Using a method previously described for isolation of mesenchymal cells from human mammary epithelial cell cultures^27^, we first derived airway epithelial Beas-2b cells that had spontaneously undergone EMT. Unlike normal epithelial-like Beas-2b cells that interact tightly with each other, these isolated Beas-2b/M cells showed the spindle morphology of a fibroblast/muscle cell phenotype (Fig. 1a) and changes of classic EMT markers such as loss of E-cadherin and increases in N-cadherin, vimentin and Snail (Fig. 1b). Profiling the signature of miRNA expression using small RNA sequencing led to identification of a core subset of down-regulated epithelial cell enriched miRNAs such as miR-205, miR-200 and miR-708 family members (Fig. 1c upper panel). In contrast, markedly up-regulated miRNAs such as miR-1, miR-10a, miR-486, miR-133a and miR-615 are typically enriched in stem cells or mesenchymal cells^28^ (Fig. 1c lower-panel). The up-regulation of miR-133a was particularly interesting because previous studies had shown its up-regulation in small airway epithelium of smokers^20^ as well as Madin-Darby Canine Kidney (MDCK) epithelial cells undergoing EMT^29,30^. Results from real-time PCR indicated that the expression level of miR-133a in mesenchymal-like Beas-2b/M cells was 10-fold higher than that of normal epithelial-like Beas-2b cells (Fig. 1d). Since non-tumor transformed lung epithelial cells will undergo type II EMT and eventually transform into fibroblast cells, we speculated that miR-133a would be highly expressed in fibroblast cells. Indeed, real-time PCR results confirmed that miR-133a expression levels in two human pulmonary fibroblast cell lines are 8- and 12-fold higher than that in airway epithelial Beas-2b cells (Supplemental Fig. S1).

### MiR-133a is up-regulated in airway epithelial cells isolated from CS exposed mice with airway hyper-responsiveness

**Figure 2:**
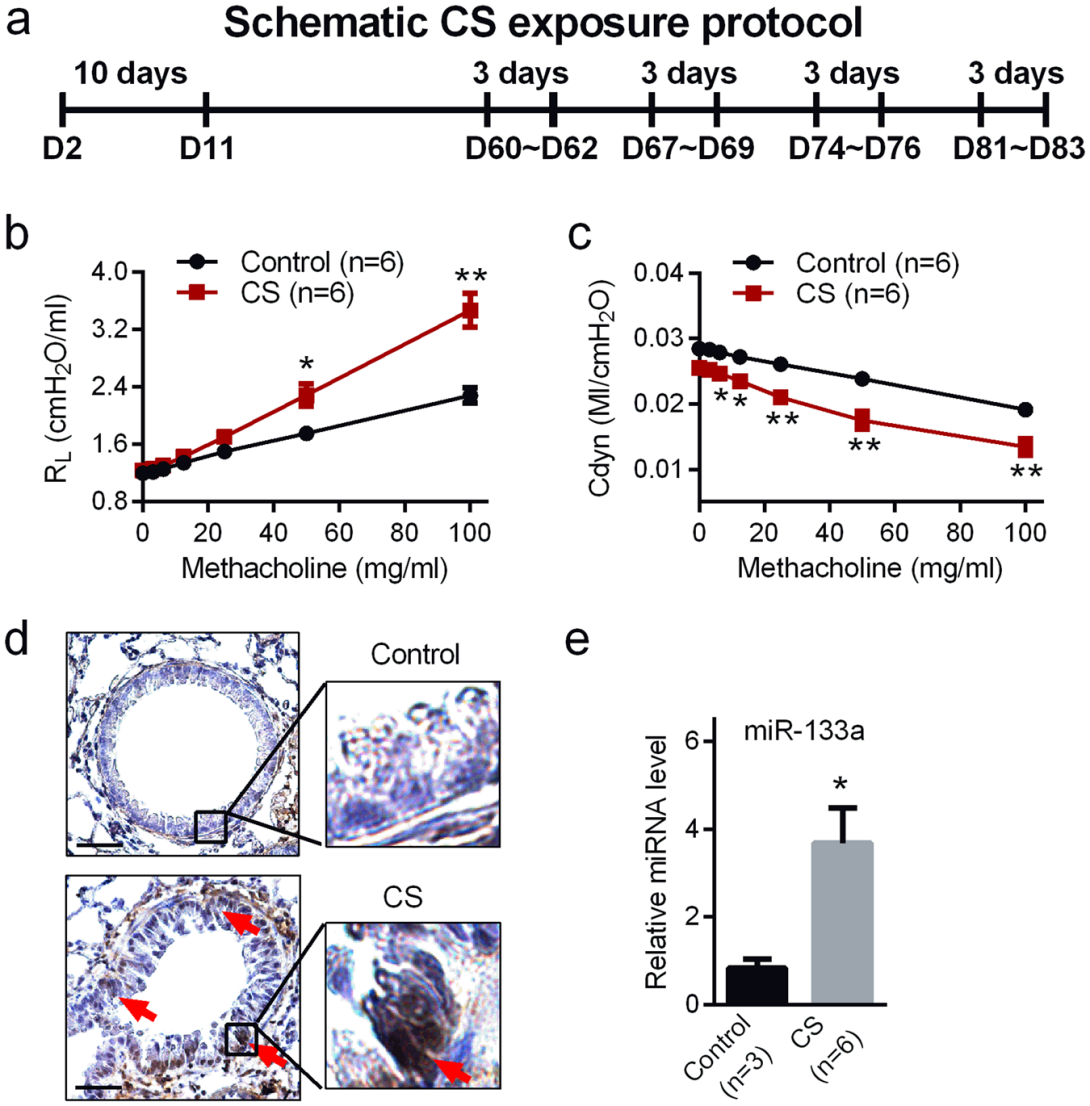
MiR-133a is up-regulated in airway epithelial cells from a cigarette smoke (CS) exposure mouse model with airway hyper-responsiveness. (**a**) Experimental scheme of CS exposure in mice. (**b**,**c**) Airway hyper-responsiveness measurements were conducted on postnatal day 84 (PND84) using invasive tracheostomy. Changes in lung resistance (R_L_) (**b**) and dynamic compliance (Cdyn) (**c**) in response to different doses of aerosolized methacholine were measured in control or CS treated mice (n = 6). (**d**) Representative N-cadherin staining of airways in mice without (Control) or with CS exposure. Red arrows indicate positive staining epithelial cells. Scale bars, 250 µm. (**e**) Tracheal cells from control and CS treated mice were isolated on PND84 and the expression level of miR-133a in these cells was validated by TaqMan quantitative PCR. Sno-202 was used as an internal control. Data are mean ± S.E. with *P < 0.05 and **P < 0.01.

CS exposure is a well-recognized risk factor for acute and chronic respiratory disease. Since exposure to environmental CS in childhood has been associated with several lung diseases, including asthma and COPD^31,32^, we established a mouse model of early-life CS exposure-induced airway hyper-responsiveness, using a protocol similar to a previous report^33^. Neonatal mice were exposed to air (control) or CS for 10 consecutive days beginning on postnatal day 2 (PND2). After recovery (PND12–59), mice were re-exposed to CS for an additional four weeks (Fig. 2a). Lung function analysis indicated that CS-exposed mice showed airway hyper-responsiveness relative to control mice, reflected by higher lung resistance (Fig. 2b) and lower dynamic compliance (Fig. 2c) in response to aerosolized methacholine, which is typical of both asthma and COPD^25^. Importantly, N-cadherin, a marker up-regulated during EMT, was positively stained in airway epithelium of CS-exposed but not in control mice (Fig. 2d). MiR-133a from tracheal epithelial cells isolated from CS exposed mice was up-regulated by 3.5-fold relative to that in these cells isolated from control mice (Fig. 2e).

### Up-regulated miR-133a induces EMT in airway epithelial cells

**Figure 3:**
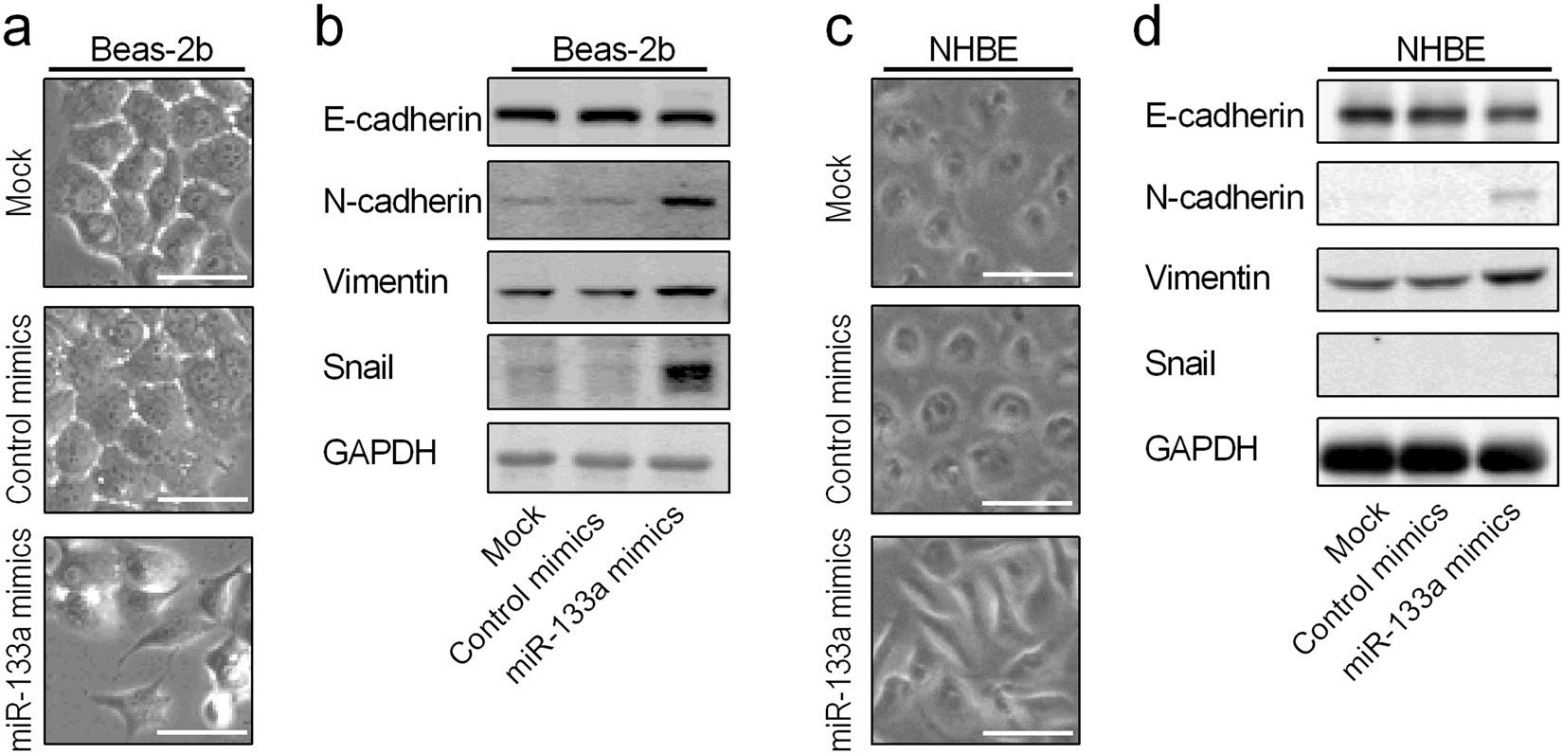
Overexpression of miR-133a in airway epithelial cells induces EMT. (**a**,**b**) Beas-2b cells were mock transfected or transfected with control or miR-133a mimics and cultured in full growth medium for 5 days. Cells were imaged with an inverted microscope (**a**) and then harvested for western blot analysis of indicated proteins (**b**). (**c**,**d**) NHBE cells were mock transfected or transfected with control or miR-133a mimics and cultured in full growth medium for 3 days before imaging and western blot analysis of indicated proteins. Experiments were conducted at least three times, and representative results are shown. Each group of blots in (**b**) and (**d**) was cropped from different parts of the same gel. Unprocessed original scans of the blots are shown in Supplementary Fig. S5. Data are mean ± S.E. with *P < 0.05. Scale bars, 100 µm.

Epithelial Beas-2b cells transfected with miR-133a mimics, but not control miRNA, showed loose cell-cell connections and a spindle cell shape typical of EMT (Fig. 3a). That miR-133a overexpression could drive EMT was confirmed by western blots showing expected changes in EMT markers (Fig. 3b). EMT driven by miR-133a overexpression was also observed in normal human bronchial epithelial cells (NHBE) as indicated by changes of cell morphology (Fig. 3c) as well as loss of E-cadherin and increases in N-cadherin and vimentin (Fig. 3d). However, there was no induction of Snail protein following miR-133a overexpression in NHBE cells.

### MiR-133a down-regulates ESRP1 protein and induces p120ctn isoform switching in airway epithelial cells

**Figure 4:**
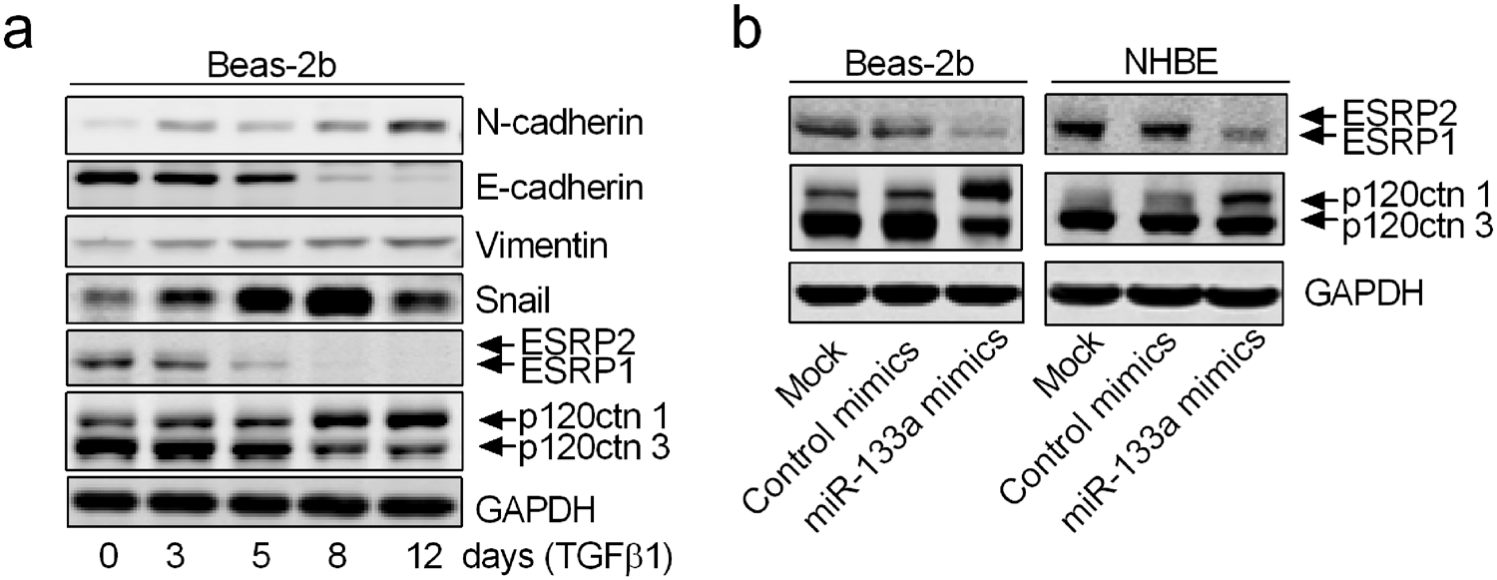
TGFβ1 and miR-133a target ESRP1 pathways in airway epithelial cells. (**a**) Beas-2b cells were treated with 5 ng/ml TGFβ1 for the indicated time. (**b**) Beas-2b and NHBE cells were mock transfected, or transfected with control or miR-133a mimics. Cells were harvested 3 days later for western blot analysis of indicated proteins. Experiments were conducted at least three times, and representative results are shown. The grouped blots in (**a**) were cropped from different parts of the same gel. The anti-p120ctn and anti-GAPDH blots in (**b**) were cropped from different parts of the same gel. However, the anti-ESRP1/2 blot was from the same sample but a different gel. Unprocessed original scans of the blots are shown in Supplementary Fig. S5.

Alternative splicing at the RNA level has emerged as critical in the regulation of EMT^34^. ESRP1/2 alters hundreds of transcripts, including isoform switching of p120ctn, a molecule with an isoform known to stabilize cadherin expression on the cell surface and help cluster cadherin in adherens junctions^35,36^. We found that ESRP1, but not ESRP2, was highly expressed in untreated epithelial Beas-2b cells and that treatment with TGFβ1 led to a gradual loss of ESRP1, along with the expected changes in classic EMT markers (Fig. 4a). Consistent with ESRP1 down-regulation, treatment of Beas-2b cells with TGFβ1 caused switching of p120ctn from isoform 3, the predominant isoform in epithelial cells, to isoform 1 (Fig. 4b), predominantly expressed in mesenchymal cells^37^. Overexpression of exogenous ESRP1 attenuated TGFβ1-induced p120ctn 1/3 isoform switch in Beas-2b cells (Supplemental Fig. S2). Interestingly, overexpression of miR-133a mimics in Beas-2b and NHBE cells also resulted in significant down-regulation of ESRP1 and the p120ctn 1/3 isoform switch (Fig. 4b). Since ESRP1 has been implicated as a central coordinator of alternative splicing networks during EMT progression, it was possible that miR-133a down-regulated ESRP1 to induce EMT in airway epithelial cells.

### MiR-133a directly targets GRHL2 expression in airway epithelial cells

**Figure 5:**
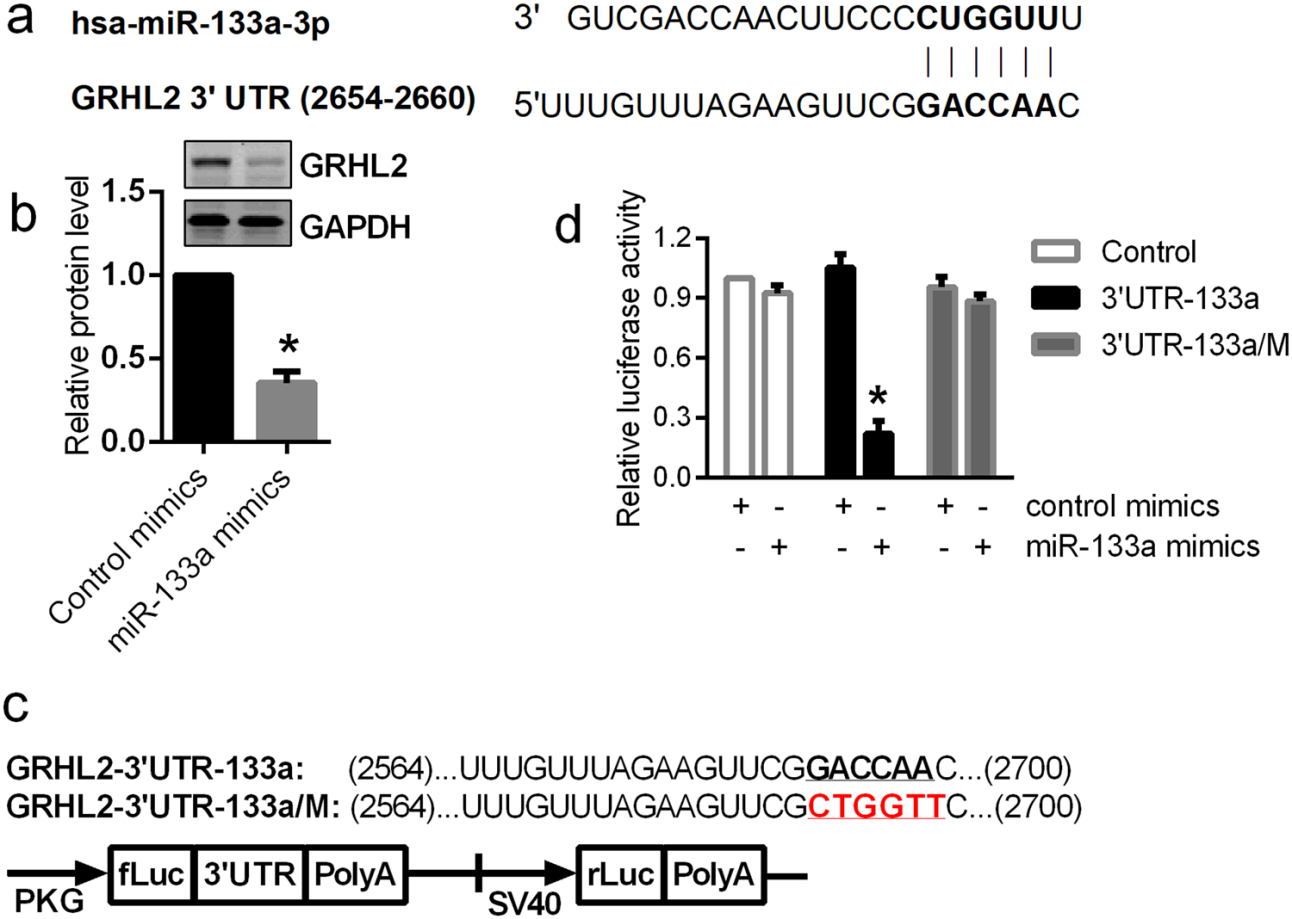
miR-133a directly down-regulates GRHL2 expression. (**a**) The 3′UTR of GRHL2 contains a putative miR-133a binding site. (**b**) Beas-2b cells were mock transfected or transfected with control or miR-133a mimics and cultured for 3 days. Cells were harvested for western blot analysis of GRHL2 protein and GAPDH was used as an internal control. Experiments were conducted at least three times, and a representative result is shown. The blots in (**b**) were cropped from different parts of the same gel. Unprocessed original scans of the blots are shown in Supplementary Fig. S5. (**c**) The 3′UTR of GRHL2 and its mutant (3′UTR-133a/M) were cloned into the pmirGLO vector and then transfected with control or miR-133a mimics into HEK293 cells. (**d**) Cells were harvested for firefly luciferase (fLuc) assays with Renilla luciferase (rLuc) as internal control. The relative luciferase activity in cells transfected with pmirGLO control vector and control mimics were normalized to 1. Data are mean ± S.E. (n = 3) with **P < 0.01.

MiRNA Target Searcher (http://www.targetscan.org/) did not identify any targeting sites for miR-133a in the 3′UTRs of ESRP1. However, the miRWalk database (http://mirwalk.uni-hd.de/)^38^ suggested that there might be a single miR-133a binding site located between 679–718 bp of the 3′UTRs of ESRP1. This predicted binding site has one nucleotide mismatching the seed sequence of miR-133a (Supplemental Fig. S3a). We cloned and inserted this 3′UTR fragment (633~807 bp) into the pmirGLO dual-luciferase reporter plasmid (Supplemental Fig. S3b). The luciferase reporter assays showed that there was no difference in the luciferase activity between cells transfected with control miRNA and the miR-133a mimic (Supplemental Fig. S3c), indicating that ESRP1 is not a direct target of miR-133a. Therefore, we focused on potential epithelial specific transcriptional factors that regulate ESRP1 expression, and identified human transcriptional factor GRHL2 as a putative target of miR-133a since its 3′UTR has a predicted miR-133a binding site (Fig. 5a). Indeed, there was a 70% reduction of GRHL2 protein in miR-133a transfected Beas-2b cells (Fig. 5b). To determine whether GRHL2 is a direct target of miR-133a, we inserted the fragment of GRHL2 3′UTR (2564–2700) containing the miR-133a target site (UTR-133a) or its mutant into the pmirGLO dual-luciferase reporter vector (Fig. 5c). Compared to control miRNA, the miR-133a mimic reduced the luciferase activity by 70% (P < 0.01). Mutation at its seed region (UTR-133a/M) completely abolished this inhibition (Fig. 5d), indicating that GRHL2 is a direct target of miR-133a. Notably, without insertion of the GRHL2 3′UTR (Control) there was no difference in the luciferase activity between cells transfected with miR-133a or its control miRNA (Fig. 5d).

### Loss of GRHL2 down-regulates ESRP1 and induces EMT in airway epithelial cells

GRHL2 is an epithelial transcriptional factor that binds to promoter or intronic enhancers of many epithelial specific genes including E-cadherin and ESRP1^39,40^. Two GRHL2 ChIP-seq data sets independently showed a potential GRHL2 binding peak in intron 4 of the *ESRP1* gene (Supplemental Fig. S4a)^40,41^. We performed chromatin immunoprecipitation (ChIP) assays to determine whether GRHL2 binds to this region of the *ESRP1 gene* in Beas-2b cells. Since we found a loss of GRHL2 and ESRP1 proteins expression in association with the p120ctn 1/3 isoform switch in mesenchymal-like Beas-2b/M cells (Supplemental Fig. S4b), Beas-2b/M cells were used as a negative control in our ChIP assays. In addition, we used the *claudin 4* (*CLDN4*) gene that contains a GRHL2 binding motif to validate our ChIP assays^40^. As shown in Supplemental Fig. S4c, in the ChIP of Beas-2b cells, we detected bands of *CLDN4* and *ESRP1* in the GRHL2 antibody group, but not in the negative control (NC) IgG group. In contrast, no bands of *CLDN4* and *ESRP1* were detected in the GRHL2 antibody group from the ChIP of Beas-2b/M cells, presumably due to loss of GRHL2 protein.

**Figure 6:**
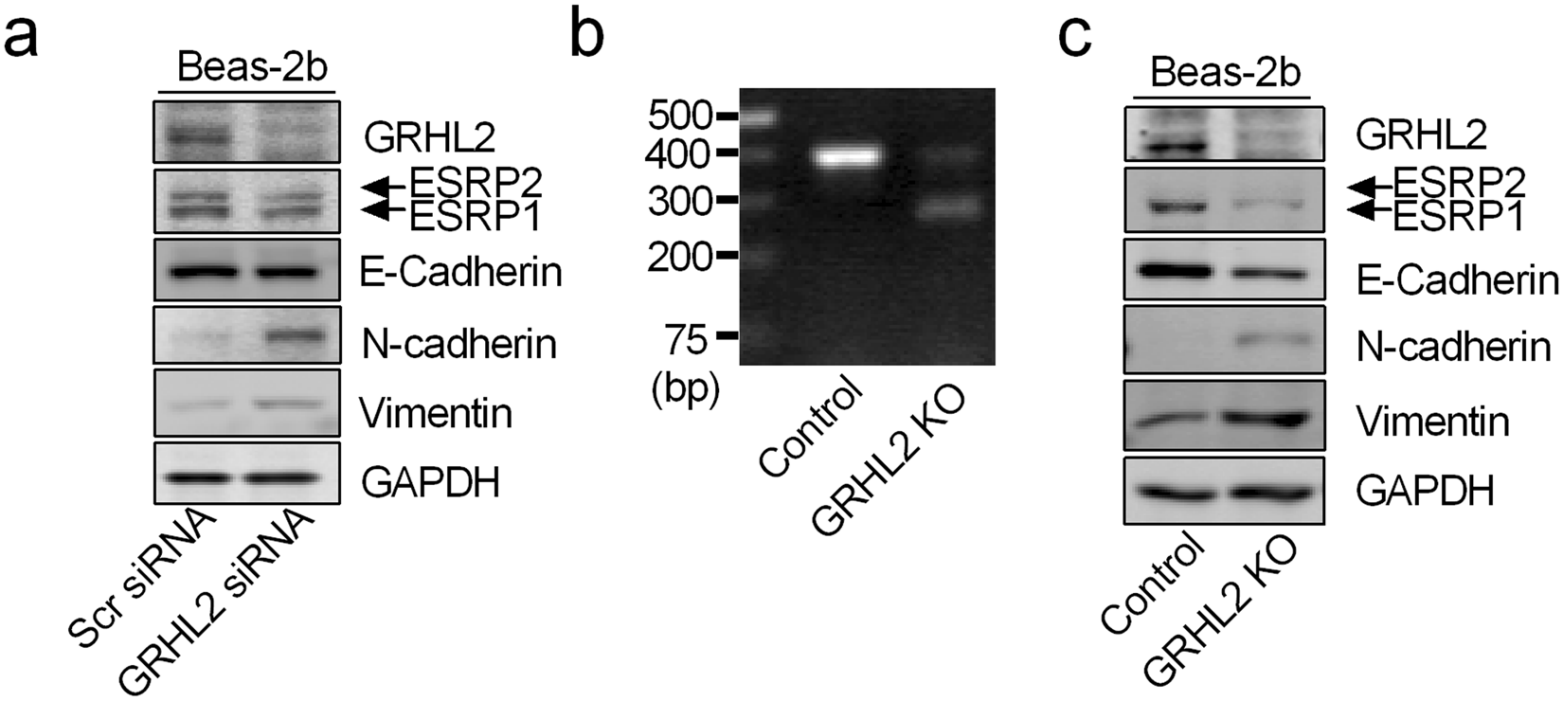
Loss of GRHL2 down-regulates ESRP1 expression and induces EMT in airway epithelial cells. (**a**) Beas-2b cells were transfected with scramble (scr) or GRHL2 siRNA for 72 hours and then harvested for western blot analysis of ERSP1 and EMT associated proteins. (**b**) Genomic DNA isolated from Beas-2b cells transfected with PX459 (control) or PX459-GRHL2g1/g2 plasmids was used as template for GRHL2 knockout (KO) confirmation. Integrated GRHL2 PCR product from the control genome was 404 bp and the PCR product from genome of PX459-GRHL2g1/g2 plasmid transfected cells has a shorter size. (**c**) Beas-2b control cells or cells with GRHL2 KO by CRISPR/Cas9 were harvested for western blot analysis of indicated proteins. Experiments were conducted at least three times, and a representative result is shown. Each group of blots in (**a**) and (**c**) was cropped from different parts of the same gel. However, the anti-GRHL2 blot was from the same sample but a different gel. Unprocessed original scans of the blots are shown in Supplementary Fig. S5.

To further determine the role of loss of GRHL2 in the development of EMT in airway epithelial cells, we used siRNAs for GRHL2 expression knockdown in Beas-2b cells. As shown in Fig. 6a, compared to scramble siRNAs, GRHL2 siRNA reduced GRHL2 protein expression by over 80%. As expected, silencing GRHL2 reduced ESRP1 expression in Beas-2b cells. More importantly, we found down-regulation of E-cadherin and up-regulation of N-cadherin and vimentin in cells transfected with GRHL2 siRNA. To confirm these results, we used the CRISPR/Cas9 technique to knockout the GRHL2 gene (Fig. 6b). As shown in Fig. 6c, GRHL2 gene deletion in Beas-2b cells down-regulated ESRP1 expression and cells underwent spontaneous EMT characterized by down-regulation of E-cadherin and up-regulation of N-cadherin and vimentin (Fig. 6c). Hence, loss of GRHL2 is an important EMT inducer in airway epithelial cells.

## Discussion

MicroRNA functions as an important regulator of EMT progression^1,10^. Complementary *in vitro* and *in vivo* approaches were employed in these studies to probe the pathophysiological mechanisms by which alterations in miRNA expression can induce EMT in epithelial cells. Rather than beginning with an arbitrarily induced *in vitro* model of EMT, we first established an airway epithelial cell line from Beas-2b cells that had spontaneously undergone EMT. Serial enrichment of poorly adherent cells in Beas-2b cell culture resulted in a cell line that we have designated Beas-2b/M due to its mesenchymal phenotype. Evidence supporting EMT includes data showing that Beas-2b/M has a fibroblast/muscle cell phenotype, up-regulation of Snail, vimentin and N-cadherin, and minimal expression of E-cadherin when compared to typical Beas-2b cells. Examination of changes in miRNA by RNA sequencing was also consistent with these results. For example, miR-205 family members known to be important for epithelial biogenesis and maintenance were almost completely lost in Beas-2b/M cells as were the miR-200 family members known to directly suppress EMT^9^.

However, the importance of miRNAs up-regulated in EMT-derived mesenchymal cells has yet to be elucidated. In this study, we focused on miR-133a based on our miRNA sequencing data and the literature. We compared the up-regulated miRNAs in our miRNA sequencing data of airway epithelial cells undergoing EMT with the up-regulated miRNAs in smoker’s airway epithelial cells^20^. Several miRNAs are up-regulated in each of the data sets, but miR-133a is the only one found in the top ten most up-regulated miRNAs of both data sets. In addition, MDCK cells have been used extensively for studies of EMT, and canine miR-133a was among the miRNAs that were highly and significantly up-regulated in the MDCK-Ras subline that underwent EMT^29^. Interestingly, miR-133a is down-regulated in metastatic prostate cancer cells and is thought to be a tumor suppressor^42^, suggesting that miR-133a up-regulation may be a component of type II rather than type III EMT. Indeed, transfection with miR-133a in airway epithelial Beas-2b and NHBE cells caused cells to display a mesenchymal cell shape typical of EMT with accompanying changes of classic EMT marker proteins.

*In vitro* studies have demonstrated that CS or its extract directly induce EMT in airway epithelial cells^5,43^. Thus, we postulated that miR-133a could be an important trigger for the EMT that gives rise to the peribronchiolar fibrosis of CS-related lung disease. Since exposure to CS in childhood has been associated with several lung diseases, including asthma and COPD^31,32^, we established a mouse model of early-life CS exposure. Our data indicate that early-life CS exposure caused mouse airways to become hyper-responsive to methacholine challenge in association with decreased compliance indicative of wall thickening and/or fibrosis. Immunohistochemical analysis provided evidence of EMT in the airways and qPCR data confirmed that miR-133a levels were increased by 3.5-fold. Thus, the data from this mouse model are qualitatively similar to those obtained from humans exposed to CS and whom are at much greater risk for developing CS-related lung disease. However, the exact mechanisms for promoting this expression of miR-133a in airway epithelial cells remains to be elucidated. Inhibition of HDAC activity is known to increase miR-133a expression^44^. Interestingly, CS exposure has been reported to significantly reduce HDAC expression and activity in cells^45,46^. Thus, it is possible that down-regulation of HADC expression and/or activity may contribute to miR-133a up-regulation in airway epithelial cells exposed to CS.

Alternative splicing has emerged as critical in the regulation of EMT^34^. Gene expression is modulated at the mRNA level via multiple RNA splicing factors to generate different isoforms of the protein^34^. ESRP1/2 are epithelial cell-specific RNA-splicing factors that regulate the production of protein isoforms associated with the epithelial phenotype, and both are down-regulated during EMT^37,47^. Both ESRP1 and ESRP2 bind to a UG-rich motif, but ESRP1 appears to be more active than ESRP2 as a splicing factor^37^. We found that ESRP1 was highly expressed in Beas-2b cells as compared to ESRP2, and that treatment with the pro-inflammatory factor TGFβ1 led to its gradual loss along with changes in classic EMT markers. Since TGFβ1 is known to induce EMT in epithelial cells derived from a wide variety of origins including airways, our data suggest that loss of ESRP1 may be a common link. To examine the potential mechanisms by which miR-133a could induce airway EMT, we examined the effects of miR-133a on expression of ESRP1 protein in airway epithelial cells. Our data showed that transfection of miR-133a into Beas-2b and NHBE cells resulted in significant down-regulation of ESRP1. We further examined the effects of miR-133a on p120ctn, a well-known ESRP1-regulated protein. P120ctn is an adherens junction-associated protein that controls E-cadherin function and stability. Loss or dissociation of p120ctn from the cadherin complex at cell junctions results in E-cadherin internalization and degradation^35,36^. P120ctn isoforms are expressed in cells via alternative RNA splicing, which is regulated by ESRPs^37^. We found that upregulation of miR-133a caused switching of p120ctn from isoform 3, the predominant isoform in epithelial cells, to isoform 1, predominantly expressed in mesenchymal cells^37^, which was accompanied by loss of E-cadherin, a hallmark of EMT. Interestingly, treatment with TGFβ1 also induced a p120ctn 1/3 isoform switch in Beas-2b cells, which could be attenuated by overexpression of exogenous ESRP1. This suggested that treatment with TGFβ1 or up-regulation of miR-133a could down-regulate ESRP1 to induce EMT via isoform switching of p120ctn as well as other genes that are involved in the EMT process.

However, the mechanism for EPSP1 down-regulation was still unclear. MiRNA Target Searcher analysis indicated that EPSP1 is not a putative direct target of miR-133a, but the miRWalk database suggested that there might be miR-133a/ESRP1 interaction. Our data from a luciferase reporter assay indicate that ESRP1 is not a direct target of miR-133a. Searching for an indirect means by which miR-133a could cause decreased expression of EPSP1 revealed that the epithelial transcription factor GRHL2 is a likely target of miR-133a. Indeed, overexpression of miR-133a caused reduction of GRHL2 protein levels by 70% and a luciferase reporter gene assay confirmed that the 3′UTR of GRHL2 is a target of miR-133a. Other studies have shown that GRHL2 is important for the establishment and maintenance of human airway epithelium, and EPSP1 is among its numerous gene targets^39,40^. A ChIP assay confirmed that GRHL2 binds to intron 4 region of the *ESRP1* gene in Beas-2b cells. In contrast, Beas-2b/M cells lost GRHL2 expression and no interaction of GRHL2 protein with the *ESRP1* gene was detected. This provides a mechanism underlying the loss of ESRP1 expression and the p120ctn 1/3 isoform switch seen in Beas-2b/M cells. Furthermore, consistent with previous observations in other epithelial cells^40,41^, knockdown of GRHL2 with siRNA or knockout of GRHL2 with CRISPR/Cas9 in Beas-2b cells is sufficient to down-regulate ESRP1 expression and cause these cells to undergo EMT. Hence, loss of GRHL2 by abnormal expression of miR-133a in airway epithelial cells could alter multiple proteins maintaining the epithelial phenotype and leads to cell EMT progression.

**Figure 7:**
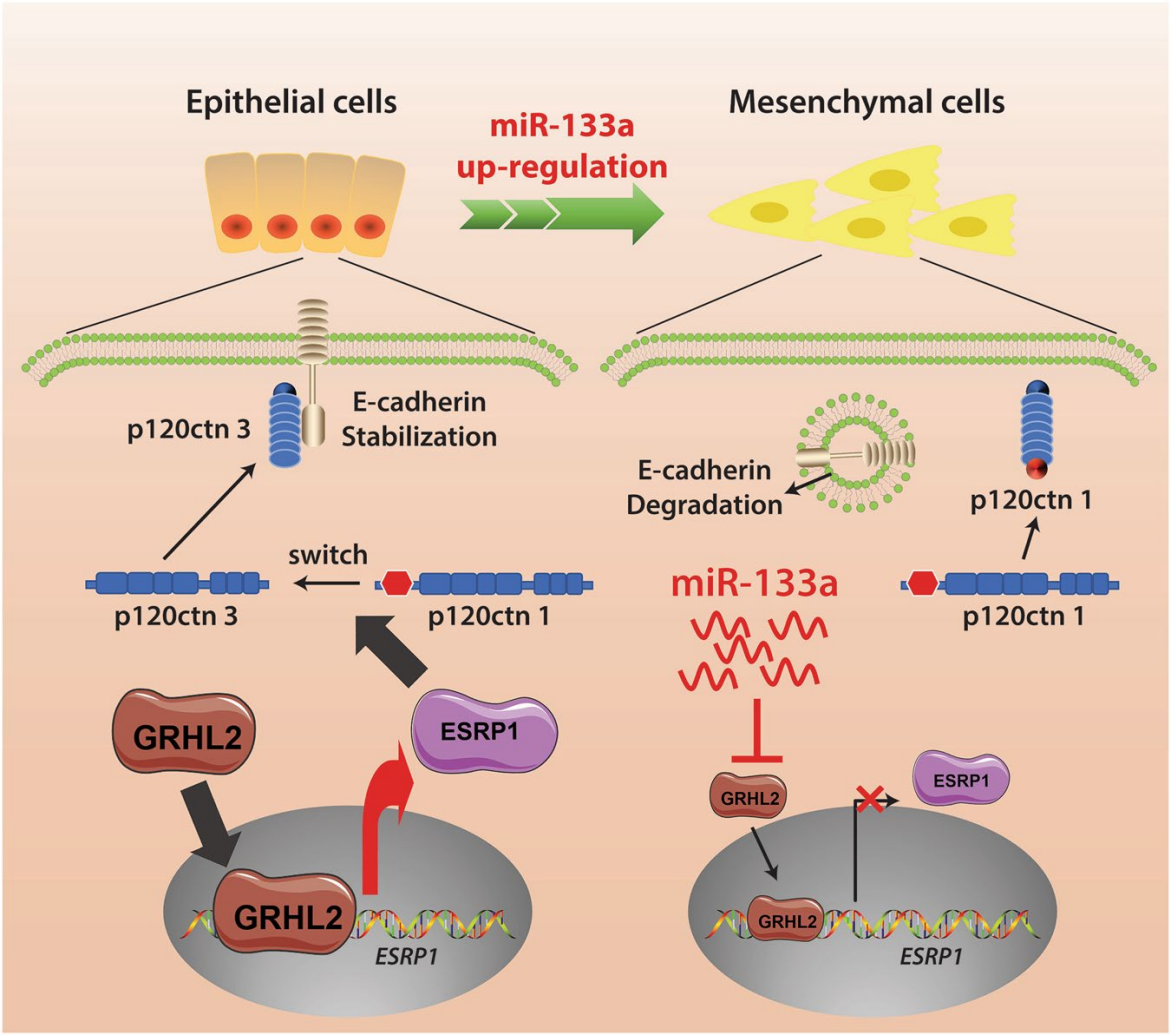
Graphical summary of data. Normal airway epithelial cells have little miR133a and abundant expression of transcription factor GRHL2, which drives the expression of ESRP1. ESRP1-mediated splicing of p120ctn pre-mRNA gives rise to p120ctn 3 mRNA. After translation, this p120ctn 3 isoform stabilizes the E-cadherin molecules that form cross linkage attachments to other nearby cells to give an epithelial cell phenotype. Up-regulated miR-133a binds directly to GRHL2 and represses its expression, which thereby represses the expression of ESRP1. With this repression of ESPR1, the p120ctn 1 mRNA is increased and translated to its protein isoform 1. Loss of p120ctn 3 leads to destabilization of E-cadherin in the plasma membrane and subsequent degradation. With sufficient levels of miR-133a, this loss of E-cadherin facilitates the transformation of airway epithelial cells into mesenchymal cells.

In conclusion, in an airway epithelial cell line in which EMT occurred spontaneously, up-regulated miR-133a was identified as a driver of this pro-fibrotic change; decreased expression of GRHL2 serves as a crucial effector (Fig. 7). Peribronchial fibrosis due to EMT is a progressive and ineffectively treated consequence of CS-related lung disease. We showed that miR-133a is up-regulated by CS exposure in a mouse model. Our data are consistent with the following paradigm for the pathogenesis of fibrotic lung disease in smokers. Chronic exposure to CS leads to persistent increases in miR-133a. At some point, a threshold for miR-133a is attained such that GRHL2 levels are inadequate to maintain the normal airway epithelial phenotype, in part due to down-regulation of EPSP1, causing EMT of damaged epithelial cells, which eventually contributes to a progressive buildup of fibrotic tissue. If this paradigm is correct, miR-133a could be an ideal target for more effective treatment of CS-related lung disease. From a drug delivery standpoint, airways can be readily targeted with reduced systemic effects by inhalation. Using a miRNA antagonism strategy such as seed-targeting 8-mer locked nucleic acid (LNA) oligonucleotides, termed tiny LNAs, may be a means of halting the pro-fibrotic process since they may be readily taken up by airway cells. Future studies geared toward elucidating the molecular basis for up-regulated miR-133a during lung disease may reveal additional targets for miR-133a inhibition.

## Electronic supplementary material

Supplementary Information

## Data Availability

All data generated or analyzed during this study are included in this published article and its Supplementary Information files.
