# Supplementary material for: Up-regulated miR-133a orchestrates epithelial-mesenchymal transition of airway epithelial cells
Source: Sci Rep. 2018 Oct 19;8:15543. doi: 10.1038/s41598-018-33913-x (PMC6195555; doi:10.1038/s41598-018-33913-x)
Supplement: Supplementary file 1 — Supplementary Information [file 41598_2018_33913_MOESM1_ESM.pdf]

Supplementary Information

Up-regulated miR-133a orchestrates epithelial-mesenchymal transition of airway epithelial cells

Linjie Chen[1], Xiaobai He[2], Yan Xie[1], Yapei Huang[1], Dennis W. Wolff[3], Peter W. Abel[1], Yaping Tu[1]

[1] Department of Pharmacology, Creighton University School of Medicine, Omaha, NE, USA

[2] College of Biotechnology, Jiangsu University of Science and Technology, Zhenjiang, Jiangsu, China

[3] Kansas City University of Medicine and Biosciences-Joplin, Joplin, MO, USA

Supplementary Methods

ChIP-PCR assay for detection of GRHL2 protein and *ESRP1* gene association

The ChIP assay was performed according to the CHIP-IT express kit manual (#53008, Active Motif North America, Carlsbad, CA) with some modifications. Briefly, 5 x 10^6 Beas-2b cells or Beas-2b/M cells were fixed for 10 min in 1% formaldehyde and scraped into the Cell Scraping Solution supplied with 1 mM phenylmethane sulfonyl fluoride. The cells were centrifuged for 10 min at 2500 rpm at 4°C, and the pellets were resuspended in a mild lysis buffer (20 mM Tris-HCl, pH 8.0, 85 mM KCl, 0.5% Nonidet P-40, protease inhibitor cocktail (Sigma)), followed by incubation on ice for 30 min. Nuclei were collected by centrifugation for 10 min at 5000 rpm, and chromatin was sheared by sonication in RIPA buffer (1× PBS, pH 7.4, 1% Nonidet P-40, 0.5% sodium deoxycholate, 0.1% SDS, protease inhibitor cocktail). GRHL2-bound chromatin fragments were immunoprecipitated with an anti-GRHL2 antibody (Sigma; HPA004820). Rabbit control IgG was used as a negative control. Cross-links were reversed by heating at 95 °C for 15 min, and DNA fragments were collected and prepared for PCR.

Two GRHL2 ChIP-seq data sets independently showed a potential binding peak of GRHL2 in intron 4 of the *ESRP1* gene[1,2]. We located a putative GRHL2 binding motif in this region (highlighted in red) (Supplemental Fig. S4a). and then designed a pair of PCR primers flanking this region (Table S1) Since we found a loss of GRHL2 and ESRP1 proteins expression in association with the p120ctn 1/3 isoform switch in mesenchymal-like Beas-2b/M cells (Supplemental Fig. S4b), Beas-2b/M cells were used as a negative control in our ChIP assays. In addition, we used the *claudin 4* (*CLDN4*) gene that contains a GRHL2 binding motif to validate our ChIP assays[1]. The PCR program was as follows: 94 ℃ for 10 minutes, then 36 cycles (94℃ for 30 seconds, 56℃ for 30 seconds and 72℃ for 30 seconds). The PCR products were analyzed on 1.5% agarose gel and the results are shown in Supplemental Fig. S4c.

References:

1. Chung, V. Y. et al. GRHL2-miR-200-ZEB1 maintains the epithelial status of ovarian cancer through transcriptional regulation and histone modification. Sci Rep 6, 19943, doi:10.1038/srep19943 (2016).

2. Gao, X. et al. Evidence for multiple roles for grainyhead-like 2 in the establishment and maintenance of human mucociliary airway epithelium. Proc Natl Acad Sci U S A 110, 9356-9361, doi:10.1073/pnas.1307589110 (2013).

Supplementary Table

Table S1. Primer pairs used in the manuscript.

pmirGLO-GRHL2	GRHL2-2564- for	5'TGTTTAAACGAGCTCaacaaagtctgaactgaacagaac
	GRHL2-2700- rev	5'CAGGTCGACTCTAGAagaaaccaattccaagatgacatc
pmirGLO-GRHL2 mutant	GRHL2-133a/M-for	5'TGTTTAGAAGTTCgctggttCAGAAAAATGCAGTCAGATG
	GRHL2-133a/M-rev	5'aaccagcGAACTTCTAAACAAACACTC
GRHL2 KO check	GRHL2-for	5'CTCCCCCATTCTGGGGAAATAA
	GRHL2-rev	5'TGGCCTGGGACAAACAATCTG
GRHL2 gDNAs	gDNA1	5'TTCAATACCCGAAGAGCCTA
	gDNA2	5'TCGGCCTGCTCTATGACTAC
ESRP1 ChIP	ESRP1-chip/for	5'GCCTTGAGGGCAGAATGAGT
	ESRP1-chip/rev	5'AGCCTCAATAGGCCAGTTTCT
CLDN4 ChIP	CLDN4-chip/for	5'GTGACCTCAGCA TGGGCTTTGA
	CLDN4-chip/rev	5' CTCCTCCTGACCAGTTTCTCTG
pmirGLO-ESRP1	ESRP1-633-for	5'TGTTTAAACGAGCTCgaaattgtgcaccctccaca
	ESRP1-807-rev	5'CAGGTCGACTCTAGAcatatgaagccacaatattcaatta

Supplementary Figures

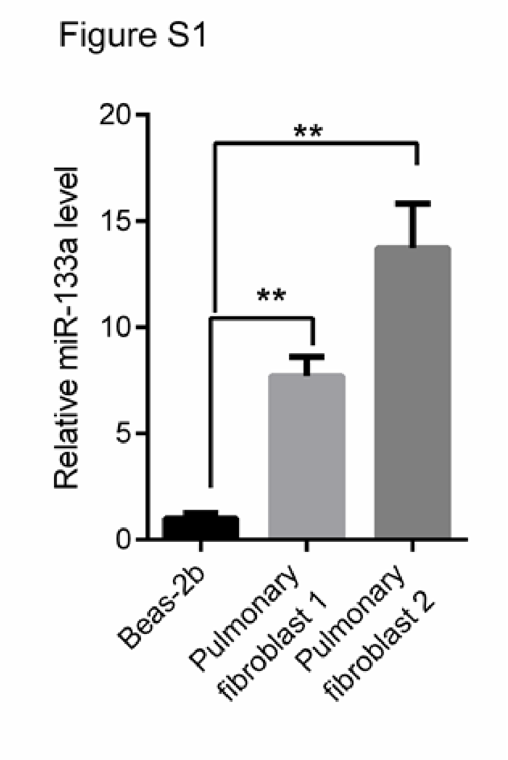

Figure S1. Pulmonary fibroblast cells have higher miR-133a expression levels. TaqMan quantitative PCR analysis showed that miR-133a is relatively highly expressed in two isolated pulmonary fibroblast cells as compared to airway epithelial Beas-2b cells. RUN6B was used as endogenous control. The relative miR-133a level in Beas-2 cells was normalized to 1. P<0.01 (n=4).

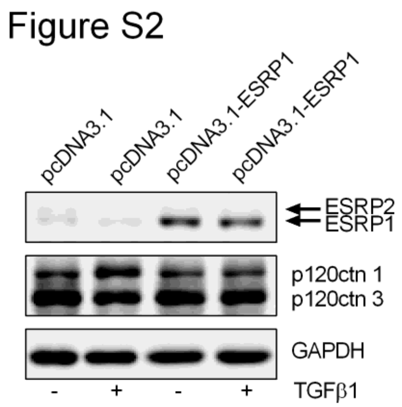

Figure S2. Overexpression of exogenous ESRP1 attenuated the p120ctn isoform switch induced by TGFβ1 treatment in Beas-2b cells. Beas-2b cells were transfected with pcDNA3.1 or pcDNA3.1-ESRP1 and selected with G418 (300 ng/ml) for 2 weeks. Cells were reseeded to a 12-well plate and treated with TGFβ1 (5 ng/ml) for 8 days before harvesting for western blot analysis. Experiments were conducted three times, and representative results are shown. The grouped blots were cropped from different parts of the same gel. Unprocessed original scans of the blots are shown in Supplementary Figure S5.

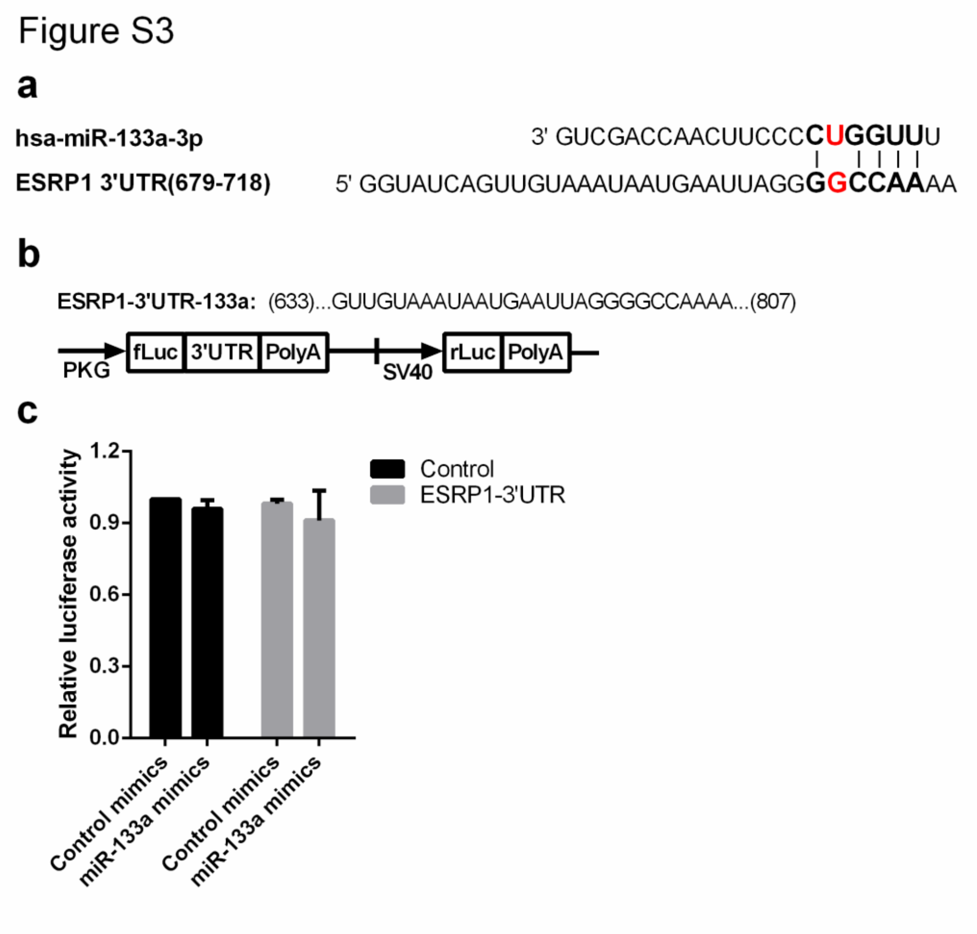

Figure S3. ESRP1 is not a direct target of miR-133a. (**a**) The putative miR-133a binding site in the 3'UTR of ESRP1 has one nucleotide mismatching the seed sequence of miR-133a. **(b)** The 3'UTR of ESRP1 (633~807bp) was cloned into the pmirGLO dual-luciferase reporter vector (ESRP1-3'UTR) and then co-transfected with control or miR-133a mimics into HEK293 cells. **(c)** Cells were harvested for firefly luciferase assays with Renilla luciferase as internal control. The relative luciferase activity in cells transfected with pmirGLO control vector and control mimics were normalized to 1. Data are mean ± S.E. (n=3).

Figure S4

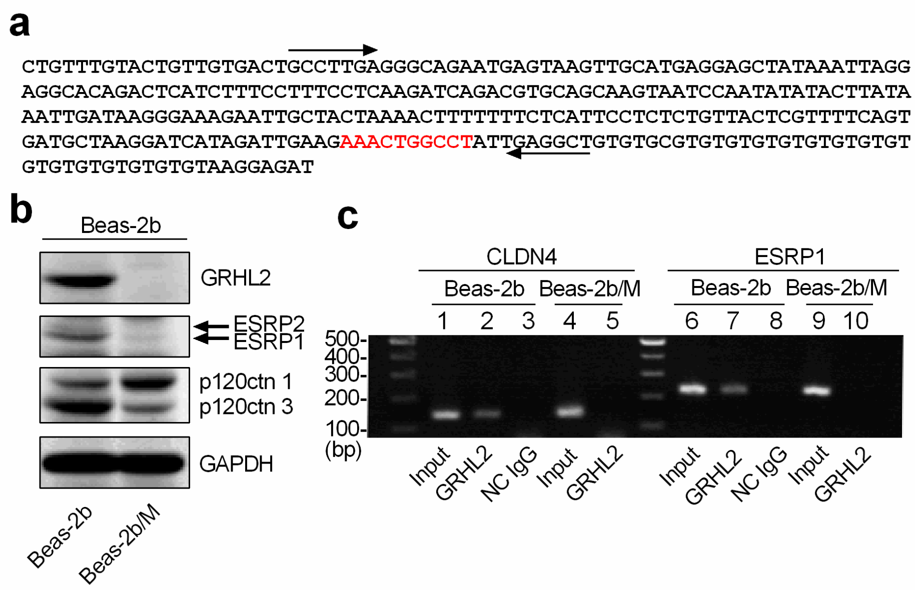

Figure S4. (a) The sequence of a potential binding peak of GRHL2 in intron 4 of the *ESRP1* gene. A putative GRHL2 binding motif in this region is highlighted in red and the arrows indicate the PCR primers for the *ESRP1* gene. **(b)** Loss of GRHL2 and ESRP1 in association with p120ctn isoform switch in mesenchymal-like Beas-2b/M cells. Experiments were conducted three times, and representative results are shown. The grouped blots were cropped from different parts of the same gel. Unprocessed original scans of the blots are shown in Supplementary Figure S5. **(c)** ChIP-PCR assays showing the binding of GRHL2 to the *CLDN4* and *ESRP1* genes. Epithelial-like Beas-2b cells and mesenchymal-like Beas-2b/M cells were fixed and scraped for ChIP-PCR assay. The predicted size of PCR products for *CLDN4* gene and *ESRP1* gene are 142 bp and 224 bp, respectively. Input, chromatin fragments before immunoprecipitation were used as PCR templates. NC IgG, negative rabbit control IgG. GRHL2, chromatin fragments immunoprecipitated with an anti-GRHL2 antibody. Experiments were repeated twice. The primers detected the *CLDN4* and *ESRP1* gene in both Beas-2b and Beas-2b/M input, respectively (lane 1, 4 and lane 6, 9). In the ChIP of Beas-2b cells, bands of *CLDN4* and *ESRP1* were detected in the GRHL2 antibody group (lane 2 and lane 7), but not in the NC IgG group (lane 3 and lane 8). In contrast, no bands of *CLDN4* and *ESRP1* were detected in the GRHL2 antibody group (lane 5 and lane 10) from the ChIP of Beas-2b/M cells.

Figure S5

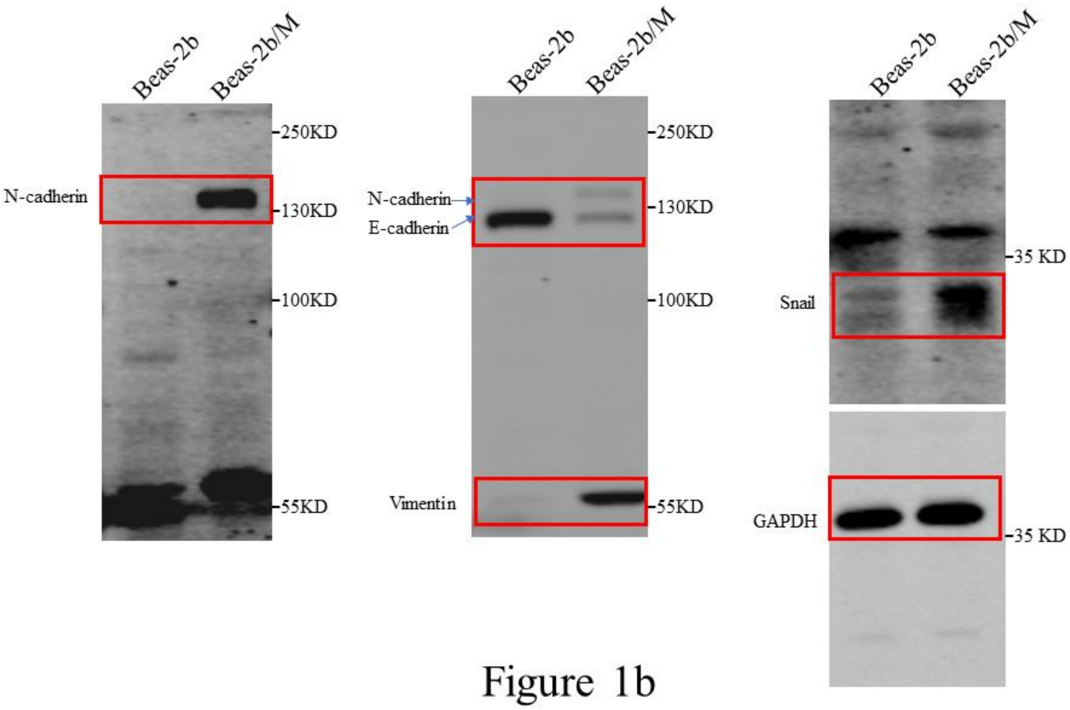

Figure 1b

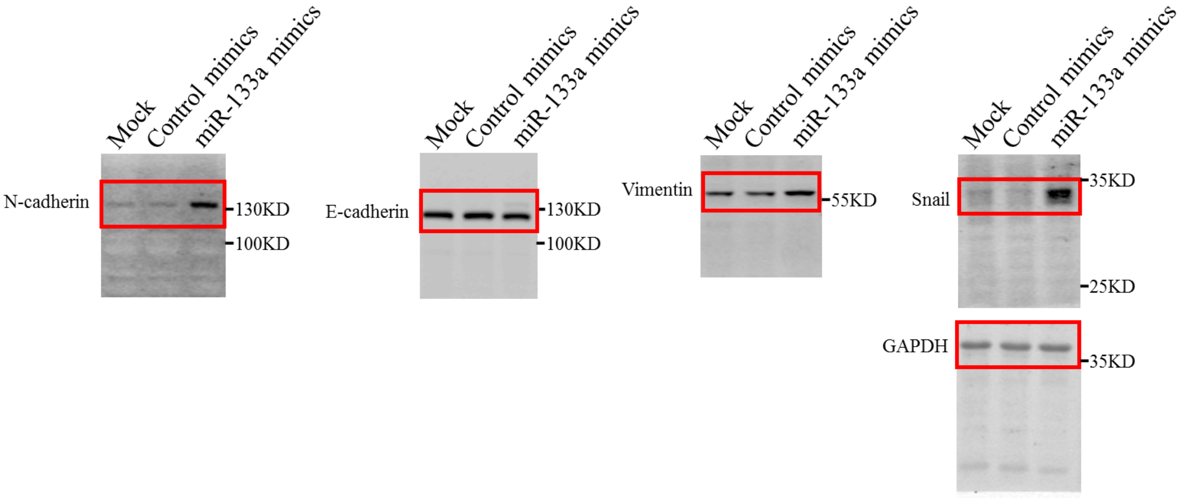

Figure 3b

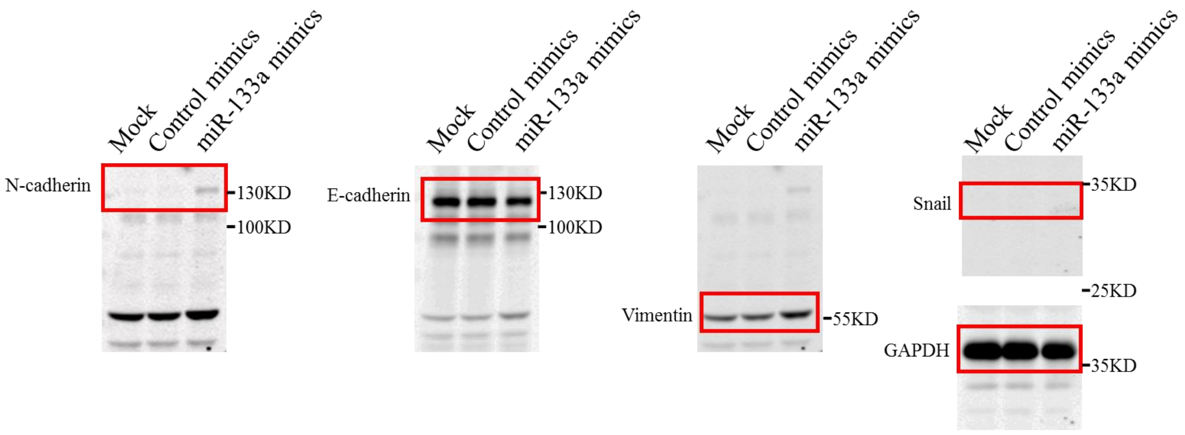

Figure 3d

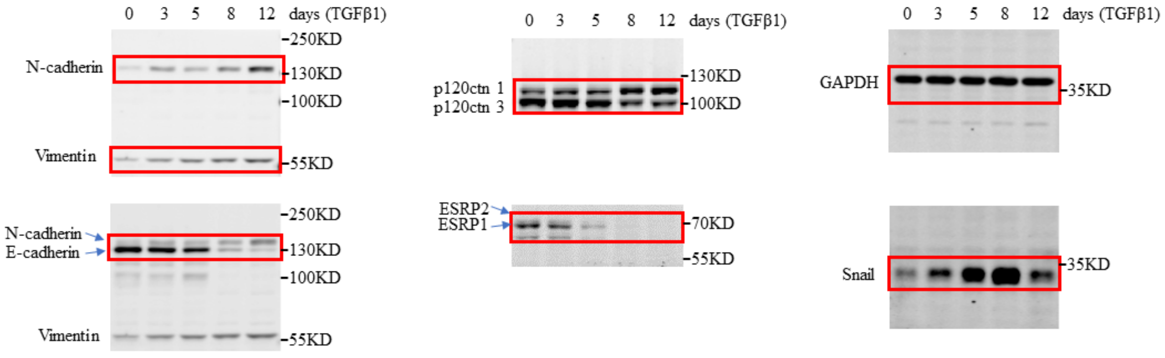

Figure 4a

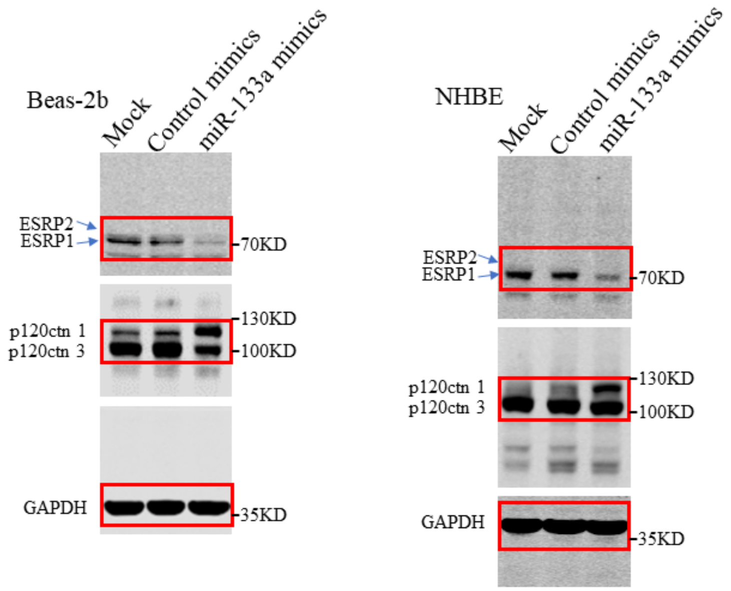

Figure 4b

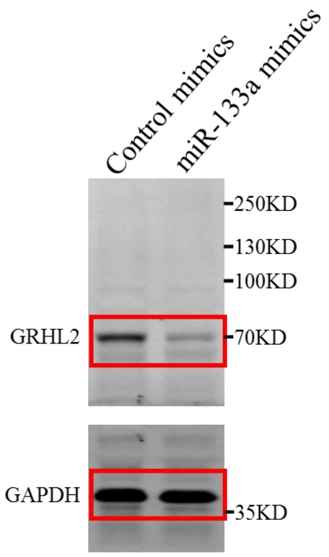

Figure 5b

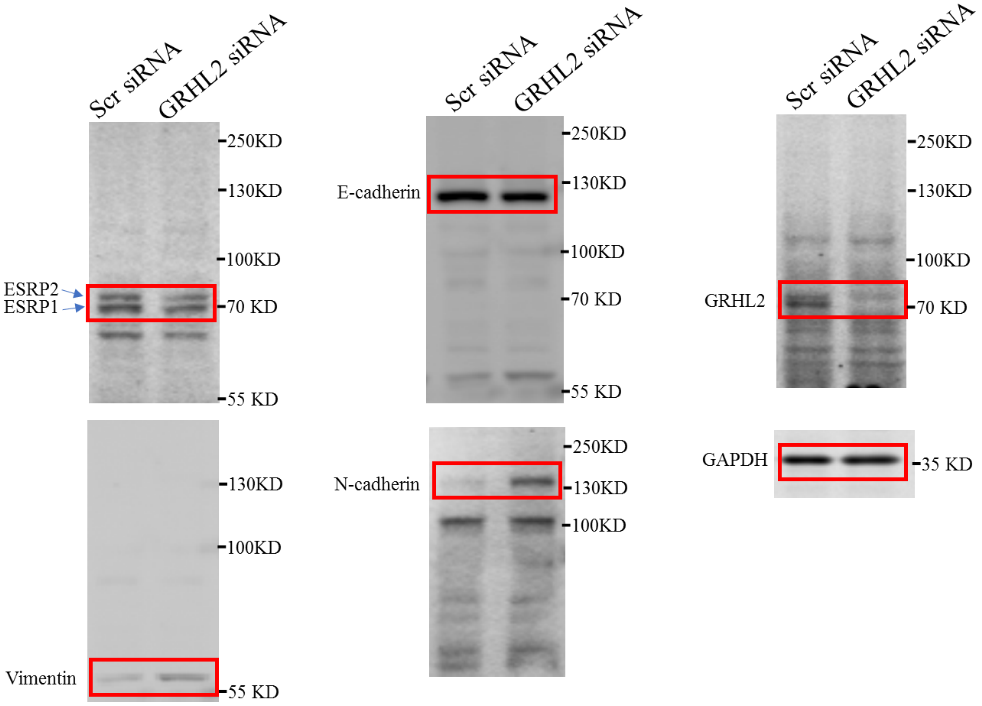

Figure 6a

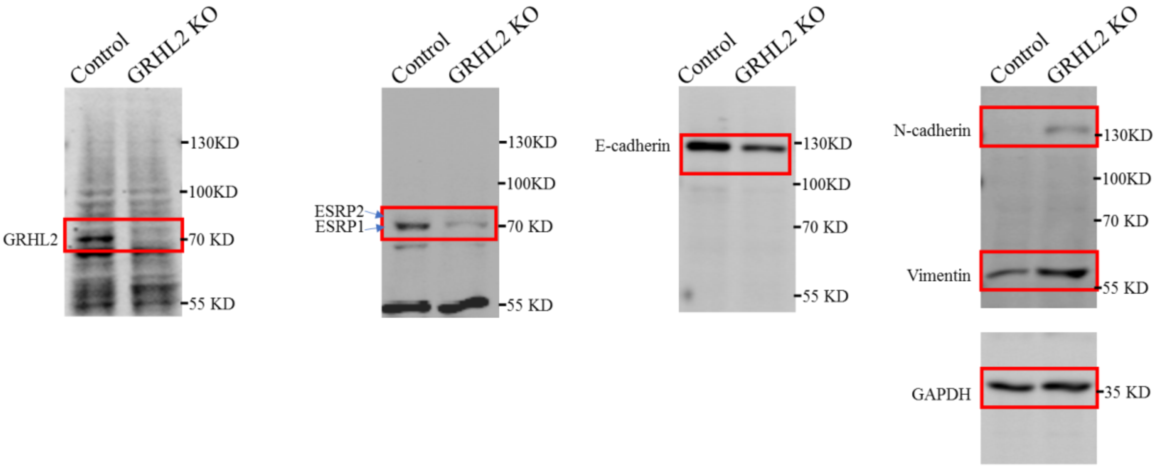

Figure 6c

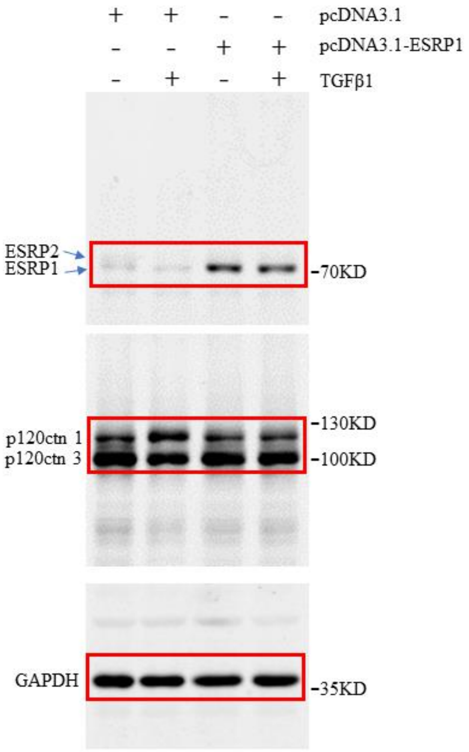

Figure S2

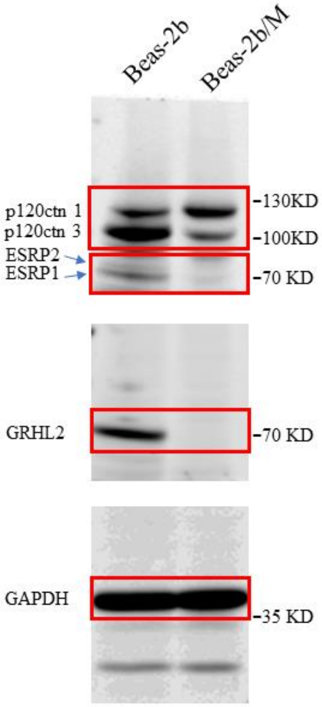

Figure S4b
